# Association between physical activity and metabolic syndrome in middle-aged Japanese: a cross-sectional study

**DOI:** 10.1186/1471-2458-11-624

**Published:** 2011-08-05

**Authors:** Junghoon Kim, Kai Tanabe, Noriko Yokoyama, Hirofumi Zempo, Shinya Kuno

**Affiliations:** 1 Department of Sports Medicine, Graduate School of Comprehensive Human Sciences, University of Tsukuba, Tsukuba, Japan; 2 Japan Society for the Promotion of Science, Tokyo, Japan; 3 Tsukuba Wellness Research. Co., Ltd., Tsukuba, Japan

## Abstract

**Background:**

Although many studies have reported an association between self-reported physical activity and metabolic syndrome (MetS), there is limited information on the optimal level of physical activity required to prevent MetS. This study aimed to determine the association between objectively measured physical activity and MetS in middle-aged Japanese individuals. We also determined the optimal cutoff value for physical activity required to decrease the risk of developing MetS.

**Methods:**

A total of 179 men and 304 women, aged between 30 and 64 years, participated in this study. Participants were divided into two groups using the Japanese criteria for MetS as those with MetS or pre-MetS, and those without MetS. Participants were considered to be physically active if they achieved a physical activity level of 23 metabolic equivalents (METs) h/week, measured using a triaxial accelerometer. The association between physical activity and MetS was analyzed using logistic regression with the following covariates: sex, age, sedentary time, low intensity activity, calorie intake, smoking, menopause and body mass index. We also evaluated the factors that determined the association between the prevalence of MetS and pre-MetS and the physical activity cutoff value using classification and regression tree (CART) analysis.

**Results:**

The odds ratio for MetS and pre-MetS was 2.20 for physically inactive participants (< 23 METs h/week), compared with physically active participants (≥ 23 METs h/week). The corresponding odds ratios for men and women were 2.27 (*P *< 0.01) and 1.95 (not significant), respectively. CART analyses revealed that moderate-vigorous physical activity of > 26.5 METs h/week was sufficient to decrease the prevalence of MetS and pre-MetS in middle-aged Japanese men and women.

**Conclusions:**

The results of this cross-sectional study indicate that the Exercise and Physical Activity Reference for Health Promotion 2006 is inversely associated with the prevalence of MetS in men. Our results also suggest that moderate physical activity of > 26.5 METs h/week may decrease the risk of developing MetS and pre-MetS in middle-aged Japanese individuals.

## Background

Metabolic syndrome (MetS) is defined as a cluster of risk factors that predispose individuals to atherosclerotic cardiovascular disease [1] and type 2 diabetes [2,3]. Individuals with MetS have higher cardiovascular disease mortality rates than do individuals without this syndrome [4,5]. Therefore, developing effective and affordable strategies to combat MetS will be of great individual and societal importance.

Current epidemiological studies indicate that moderate-vigorous physical activity (MVPA) is associated with decreased risk of developing MetS, independent of aerobic fitness and obesity [6-11]. Other studies have also shown that daily physical activity prevents the onset of chronic diseases such as type 2 diabetes and MetS [12,13]. The Exercise and Physical Activity Reference for Health Promotion report 2006 (EPAR2006) compiles the current guidelines for health promotion and gives recommendations for physical activity and exercise for Japan [14,15]. These guidelines focus on daily physical activity exceeding 3 metabolic equivalents (METs), which includes both lifestyle physical activity and exercise, unlike the former exercise guidelines [15]. According to EPAR2006, every adult should accumulate 23 METs h/week of physical activity to prevent the onset of lifestyle-related diseases [14].

The guidelines for physical activity specified in EPAR2006 were established on the basis of a systematic review of studies performed mostly in Western countries, and included very few studies measuring physical activity in Japanese individuals. The Japanese population differs from the European and American populations in terms of genetic background and social environment. Furthermore, the guidelines were largely established using data obtained from a self-reported questionnaire survey. Such analyses are prone to recall error and over-reporting, and are limited by their specific focus on MVPA [16,17]. Furthermore, it is difficult to accurately evaluate activities of daily life using a questionnaire [18]. Objective assessment of physical activity using accelerometers can overcome these limitations. This is supported by the results of a recent study that demonstrated a good construct validity for accelerometers compared with self-reported energy expenditure from physical activity [19].

Although many studies have reported an association between self-reported physical activity and MetS, there is limited information on the optimal level of physical activity required to prevent MetS and its associated cardiovascular abnormalities. Moreover, EPAR2006's physical activity parameters have not yet been correlated with MetS. Therefore, the objective of this study was to establish a physical activity reference relative to MetS in middle-aged Japanese men and women using an objective measurement of physical activity. We also determined the optimal cutoff value for physical activity required to decrease the risk for MetS.

## Methods

### Participants

The participants in this cross-sectional study were 521 healthy middle-aged Japanese volunteers without diabetes, cardiovascular disease, and musculoskeletal diseases recruited from local community newspapers in Tsukuba, Ibaraki. We excluded individuals who were missing data on physical activity (n = 11), MetS components (n = 18) or dietary intake (n = 9). After excluding these individuals, a total of 179 men and 304 women were included in this analysis [age, 30-64 years; body mass index (BMI), 17.2-45.5 kg/m^2^]. This study was approved by the Ethical Committee of the Institute of Health and Sport Sciences and the Institute of Clinical Medicine at the University of Tsukuba. Written informed consent was obtained from all participants. Participant characteristics (n = 483) are presented in Table 1.

**Table 1:** Descriptive characteristics of study participants

	Men (n = 179)	Women (n = 304)	*P *value
Age (years)	46.6 (0.6)	48.6 (0.5)	0.015
Height (cm)	170 (0.5)	157.7 (0.3)	< 0.001
Body weight (kg)	73.9 (0.9)	63.7 (0.6)	< 0.001
BMI (kg/m^2^)	25.6 (0.3)	25.6 (0.2)	0.896
% muscle mass (%)	30.4 (0.2)	23.7 (0.3)	< 0.001
% fat mass (%)	25.1 (0.4)	34.0 (0.4)	< 0.001

Physical activity parameter*			
Sedentary time (h/week)	37.2 (0.9)	29.5 (0.7)	< 0.001
Low intensity activity (METs h/week)	77.1 (1.8)	96.4 (1.3)	< 0.001
MVPA (METs h/week)	24.5 (0.8)	25.7 (0.6)	0.252

***Metabolic syndrome components***			
Abdominal obesity (%)	66.7	43.9	< 0.001
Hyperglycemia (%)	29.8	18.8	0.005
Hypertension (%)	54.2	47.7	0.168
Dyslipidemia (%)	33.5	14.5	< 0.001
Number of MetS components (n)	1.9 (0.1)	1.3 (0.1)	< 0.001

***Metabolic syndrome***			< 0.001
Pre-MetS (%)	18.4	17.8	
MetS (%)	34.6	16.4	
MetS/pre-MetS (%)	53.1	34.2	

***Daily nutritional intake***^†^			
Calorie intake (kcal/day)	2126.1 (24)	1899.1 (15.6)	< 0.001
Carbohydrate intake (g/day)	253.0 (7)	238.6 (4.8)	0.081
Protein intake (g/day)	76.9 (1.9)	68.6 (1.6)	0.010
Fat intake (g/day)	60.0 (1.8)	55.5 (1.5)	0.058

Smoker (%)	22.3	5.3	< 0.001
Postmenopausal (%)	-	52.6	

Values are means (SE) or percentage. *Adjusted for age, ^†^adjusted for body weight. BMI, body mass index; METs, metabolic equivalents; MVPA, moderate-vigorous physical activity; MetS, metabolic syndrome; SE, standard error. Abdominal obesity: waist circumference ≥ 85 cm for men and ≥ 90 cm for women; hyperglycemia: blood glucose ≥ 110 mg/dL; hypertension: systolic blood pressure ≥ 130 mmHg and/or diastolic blood pressure ≥ 85 mmHg, or treatment for previously diagnosed hypertension; dyslipidemia: triglyceride ≥ 150 mg/dL and/or high-density lipoprotein cholesterol level < 40 mg/dL, or specific treatment for these lipid abnormalities.

### Anthropometric and body composition measurements

Body weight was measured to the nearest 0.1 kg using a digital scale (TBF-551; Omron Healthcare Co., Ltd., Kyoto, Japan), and height to the nearest 0.1 cm using a wall-mounted stadiometer. BMI was calculated as body weight/height^2 ^(kg/m^2^). Waist circumference (WC) was measured three times to the nearest 0.1 cm at the midpoint between the lower costal margin and the iliac crest using a calibrated measuring tape. Body fat percentage (% body fat) and muscle mass percentage (% muscle mass) were calculated from the impedance value (HBF-352, Omron Healthcare Co., Ltd., Kyoto, Japan) [20].

### Measurement of physical activity and sedentary time

A triaxial accelerometer (HJA-350IT, Active style Pro, Omron Healthcare Co., Ltd.) was used to measure physical activity and sedentary time [21]. To ensure a valid reflection of long-term day-to-day activities, the accelerometer was required to be worn by participants for 7 days while the subjects were continuing their regular daily activities [22]. The participants were instructed to wear the accelerometer on an elastic waist band on the right hip throughout the day from the time they woke up in the morning until they went to bed at night, except during showers and bathing. Participants who did not record 10 hours/day of activity for 7 days were excluded from further analyses [22]. The mean wear time was 11.8 ± 1.6 hours/day. Accelerometer data were calculated as the monitoring time spent in each of three different intensity levels for 1 week, using software provided by the manufacturer (HMS-HJA-IC01J; Omron Healthcare Co., Ltd.) as follows: sedentary (1.1-1.4 METs), low (1.5-2.9 METs), and moderate-to-vigorous (≥ 3 METs). To compare the impact of different levels of MVPA on the risk of MetS, we defined two categories of MVPA based on the physical activity reference recommended in the EPAR2006: < 23 METs h/week and ≥ 23 METs h/week.

### Blood pressure and biochemical blood assay

Blood pressure was measured twice using an electronic digital blood pressure monitor (SM-100; Omron Healthcare Co., Ltd.) with subjects in a seated position after a 10-min rest period. If the two readings differed by more than 5 mmHg, a third reading was taken and the lowest was used. Approximately 10 mL of blood was drawn from each subject after an overnight fast. Fresh samples were used for enzymatic analysis of triglyceride levels, while fasting plasma glucose levels were assayed using the glucose oxidase method. Serum high-density lipoprotein cholesterol (HDL-C) was measured using heparin-manganese precipitation.

### Components of MetS

The Japanese criteria for MetS were used to evaluate the prevalence of MetS and pre-MetS in this study [23,24]. Based on these criteria, to diagnose MetS, the subject must present with abdominal obesity, which is a mandatory criterion because it is a strong predictor of cardiovascular risk factors, in addition to two or more of the other components. For the diagnosis of pre-MetS, the subject must have abdominal obesity and one of the other components, including (1) WC ≥ 85 cm for men and ≥ 90 cm for women, which is considered an essential component; (2) dyslipidemia (triglyceride ≥ 150 mg per dL and/or HDL-C level < 40 mg per dL, or specific treatment for these lipid abnormalities); (3) hypertension (systolic blood pressure ≥ 130 mmHg and/or diastolic blood pressure ≥ 85 mmHg, or treatment of previously diagnosed hypertension); or (4) hyperglycemia (fasting plasma glucose ≥ 110 mg/dL). In this study, we analyzed physical activity for MetS subjects and pre-MetS subjects in a single group (MetS/pre-MetS).

### Covariates

A nutritionist assessed the daily nutritional intake of each subject, including carbohydrate, fat and protein, as well as the total calorie intake per day, by evaluating the patients' 3-day weight and dietary records. The food data of the dietary records were converted to energy and nutrient data by the dietician and analyzed using Excel Eiyo-kun software (Ver. 4.0; Kenpaku Co., Ltd., Tokyo, Japan). Participants also completed a general health questionnaire, which recorded smoking and menopausal status.

### Statistical analysis

Differences in physical characteristics between subjects with MetS/pre-MetS and subjects without MetS were tested using unpaired *t*-tests. Physical activity parameters and nutrient intake were analyzed in both groups using analysis of covariance (ANCOVA) with age or body weight as covariates, respectively.

Logistic regression analysis was used to predict MetS from the levels of physical activity based on the EPAR2006 reference (≥ 23 METs h/week or < 23 METs h/week); this was done separately for men and women. In all logistic regression models, age, level of low intensity activity, sedentary time, smoking, calorie intake and BMI were included as covariates. The total cohort model was also adjusted for sex. The women's model was adjusted for menopause. Multicollinearity between sedentary time, level of low intensity activity and MVPA was tested but was not significant.

Classification and regression tree analysis (CART) [25,26] was used to establish the cutoff value for required physical activity and the best predictor for the prevalence of MetS. CART analysis is constructed by repeatedly splitting subsets of the data using all explanatory variables to create two subgroups. Explanatory variables with the best improvement are selected to split the data based on the improvement score, and are evaluated to identify the optimal cutoff point (continuous variable) or groups (nominal variables). We calculated *P *values at each branch using χ^2 ^tests. Values are expressed as means ± standard error (SE) or as percentages. Values of *P *< 0.05 were considered statistically significant. All data were analyzed using SPSS version 17 or R version 2.10.1 [27].

## Results

This study included 483 Japanese individuals (men 37.1%, women 62.9%) aged between 30 and 64 years (Table 1). Overall, 53.1% of men and 34.2% of women had MetS or pre-MetS (Table 1). The prevalence of abdominal obesity, hyperglycemia, dyslipidemia, and MetS values was significantly higher in men than in women (Table 1). MVPA was not significantly different between men and women (*P *= 0.252).

Table 2 shows the characteristics of participants with MetS/pre-MetS and of participants without MetS, by sex. In both men and women, we found significant differences in body weight and BMI between individuals with MetS/pre-MetS and those without MetS (Table 2). As described in a previous study, we assessed possible covariates that were likely to influence the prevalence of MetS. Because there seems to be a strong age-dependence in the prevalence of MetS [28], we included age as a covariate to adjust the physical activity parameters. With the inclusion of age in the model, we found a significant difference in MVPA in both men and women (Table 2). Notably, age, sedentary time and low intensity physical activity were significantly associated with MetS/pre-MetS, but only in women. In contrast, daily nutrition intake adjusted by body weight, including total energy intake, and total carbohydrate, fat and protein intake, was not different between MetS/pre-MetS and non-MetS in both men and women (Table 2).

**Table 2:** Characteristics of participants with MetS/pre-MetS and those without MetS, by sex

	Men			Women		
	
	Without MetS (n=85)	MetS/pre-MetS (n = 94)	*P *value	Without MetS (n = 200)	MetS/pre-MetS (n = 104)	*P *value
Age (years)	45.5 (0.9)	47.6 (0.8)	0.088	46.7 (0.7)	52.3 (0.8)	< 0.001
Height (cm)	168.8 (0.7)	171 (0.6)	0.023	157.8 (0.4)	157.6 (0.6)	0.853
Body weight (kg)	66.9 (0.9)	80.1 (1.1)	< 0.001	58.6 (0.5)	73.4 (0.9)	< 0.001
BMI (kg/m^2^)	23.5 (0.3)	27.4 (0.3)	< 0.001	23.6 (0.2)	29.5 (0.3)	< 0.001
% muscle mass (%)	30.8 (0.4)	30.2 (0.2)	0.102	24.8 (0.2)	21.9 (0.5)	< 0.001
% fat mass (%)	23.3 (0.6)	26.5 (0.4)	< 0.001	31.7 (0.4)	37.7 (0.4)	< 0.001

Physical activity parameters*						
Sedentary time (h/week)	36.5 (1.4)	38.2 (1.3)	0.367	27.6 (0.8)	32.9 (1.1)	0.001
Low intensity activity (METs h/week)	75.5 (2.8)	77.9 (2.6)	0.538	99.5 (1.6)	90.9 (2.2)	0.002
MVPA (METs h/week)	28.4 (1.2)	21.0 (1.2)	< 0.001	27.6 (0.7)	23 (1.0)	0.001

***Metabolic syndrome components***						
Waist circumference (cm)	82.6 (0.6)	93.4 (0.9)	< 0.001	83.1 (0.5)	100.9 (0.8)	< 0.001
SBP (mmHg)	122.7 (1.4)	136.7 (1.3)	< 0.001	118.9 (0.9)	136.4 (1.9)	< 0.001
DBP (mmHg)	79.1 (1.0)	90.5 (1.1)	< 0.001	79.1 (0.7)	91.8 (1.1)	< 0.001
Fasting glucose (mg/dL)	100.3 (1.0)	109.7 (1.6)	< 0.001	97.2 (0.8)	115.1 (3.1)	< 0.001
Triglyceride (mg/dL)	93.2 (5.7)	173.0 (9.8)	< 0.001	82.6 (3.1)	139.4 (8.3)	< 0.001
HDL-C (mg/dL)	61.7 (1.2)	53.2 (1.5)	< 0.001	67.3 (1.0)	59.1 (1.3)	< 0.001
Number of MetS components (n)	0.7 (0.1)	2.9 (0.1)	< 0.001	0.6 (0.0)	2.6 (0.1)	< 0.001

***Daily nutrition intake***^†^						
Calorie intake (kcal/day)	2122.2 (32.1)	2129.6 (35.4)	0.878	1897.8 (20.6)	1901.4 (22.6)	0.913
Carbohydrate intake (g/day)	252.7 (11.4)	253.3 (9.0)	0.971	241 (6.1)	233.6 (7.9)	0.471
Protein intake (g/day)	74.5(3.1)	78.6 (2.4)	0.292	68.9 (1.9)	67.9 (3)	0.753
Fat intake (g/day)	59.8 (2.6)	60.1 (2.4)	0.948	56.7 (1.7)	53.2 (2.9)	0.271

Smoker (%)	17.9	26.3	0.175	5.5	4.8	0.798
Postmenopausal (%)	-	-		45.0	67.3	< 0.001

Values are means (SE) or percentage. *Adjusted for age, ^†^adjusted for body weight. MetS, metabolic syndrome; BMI, body mass index; METs, metabolic equivalent; MVPA, moderate-vigorous physical activity; SBP, systolic blood pressure; DBP, diastolic blood pressure; HDL-C, high density lipoprotein cholesterol; SE, standard error.

Results of logistic regression analyses comparing the prevalence of MetS/pre-MetS across physical activity categories by sex are shown in Table 3. In all analyses, the physically active group that met the physical activity reference standard (≥ 23 METs h/week) was used as the reference group. After adjusting for sex, age, low intensity activity, sedentary time, smoking, calorie intake and BMI, the risk of MetS/pre-MetS in the physically inactive group was significantly increased compared with that in the physically active group [Table 3: odds ratio (OR), 2.20; 95% CI, 1.7-3.83]. The risk for MetS among physically inactive men was significantly higher than that for physically active men after adjustment for age, sedentary time, low intensity activity, smoking, calorie intake and BMI (Table 3: OR, 2.27; 95% CI, 1.22-6.29). In contrast, the risk for MetS in women was not significantly different between physically active and physically inactive women after adjustment for age, sedentary time, low intensity activity, smoking, calorie intake, BMI and menopausal status (Table 3: OR, 1.95; 95% CI, 0.85-4.45).

**Table 3:** Odds ratios for the prevalence of metabolic syndrome according to physical activity levels

MetS/pre-MetS	Univariate YCrude OR (95% CI)	Multivariate Adjusted OR (95% CI)
Total (n = 483)		
≥ 23 METs h/week	1	1
< 23 METs h/week	2.20 (1.52-3.19)	2.20 (1.27-3.83)*

Men (n = 179)		
≥ 23 METs h/week	1	1
< 23 METs h/week	2.27 (1.24-4.15)	2.27 (1.22-6.29)^†^

Women (n = 304)		
≥ 23 METs h/week	1	1
< 23 METs h/week	1.97 (1.22-3.20)	1.95 (0.85-4.45)^‡^

*Adjusted for sex, age, calorie intake, smoking, sedentary time, level of low intensity activity and body mass index (BMI); ^†^adjusted for age, calorie intake, smoking, sedentary time, level of low intensity activity and BMI; ^‡^adjusted for age, calorie intake, smoking, sedentary time, level of low intensity activity, BMI and menopause. OR, odds ratios; CI, confidence interval.

**Figure 1:**
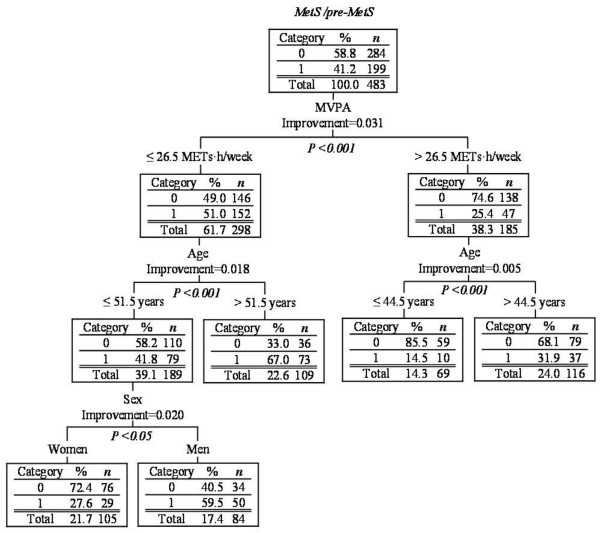
**Classification tree of participants for the prevalence of metabolic syndrome**. The participants in category 0 did not have MetS, whereas those in category 1 had MetS/pre-MetS. MetS, metabolic syndrome; METs, metabolic equivalents; MVPA, moderate-vigorous physical activity.

As shown in Figure 1, eight factors (MVPA, sedentary time, low intensity activity, sex, age, smoking, calorie intake and menopausal status) were included in the CART analysis. The first table in Figure 1 shows that MVPA decreased the prevalence of MetS/pre-MetS in 199 of the 483 participants (41.2%). The most important factor that decreased the prevalence of MetS/pre-MetS was an MVPA of > 26.5 METs h/week (*P *< 0.001). The prevalence of MetS/pre-MetS was lower in 47 of the 185 participants with an MVPA of > 26.5 METs h/week (25.4%) and in 152 of the 298 participants (51.0%) with an MVPA of ≤ 26.5 METs h/week. Age was used to subdivide the participants, regardless of whether activity of 26.5 METs h/week was achieved. In the subgroup with an MVPA of > 26.5 METs h/week, the prevalence of MetS/pre-MetS was decreased in 10 of the 69 participants aged ≤ 44.5 years (14.5%) and in 37 of the 116 participants aged > 44.5 years (31.9%). In the ≤ 26.5 METs h/week group, age was also the most important factor for the split, with increased prevalence of MetS/pre-MetS in 79 of 189 (41.8%) and in 73 of 109 (67.0%) participants aged ≤ 51.5 years and > 51.5 years, respectively. Sedentary time, low intensity activity, smoking, calorie intake and menopausal status were not significant parameters in CART analysis.

## Discussion

This study aimed to determine a physical activity reference value relative to MetS in middle-aged Japanese men and women, to establish an appropriate cutoff value for physical activity that, if met, will decrease the prevalence of MetS. The results suggest that the EPAR2006 physical activity reference was significantly associated with MetS in men but not in women after adjustment for covariates. Moreover, we found that the cutoff value of physical activity required to decrease the risk of MetS was 26.5 METs h/week in middle-aged Japanese individuals.

### Association between physical activity levels specified by EPAR2006 and MetS

This present study is the first, to our knowledge, to examine the association between the EPAR2006-recommended physical activity levels and MetS. The analysis showed that physically active men (≥ 23 METs h/week) were at less risk of MetS than their physically inactive counterparts (< 23 METs h/week). In contrast, the association between MVPA and MetS in women was less clear. Our physical activity findings were similar to those reported by other similarly designed studies [6-8]. Contemporary analyses have shown that leisure-time physical activity is effective in preventing and treating MetS [7,29], and most studies have shown that MetS is inversely associated with physical activity or physical fitness in men [6,30,31]. In contrast, few studies have observed inverse associations between physical activity and MetS in women [32].

Our study confirms the results of other studies showing an inverse association between physical activity and MetS, which applies particularly to men. Zhu et al. [33] reported a significant association between physical activity and MetS risk factors in men, but not in women, after considering covariates like age, race, income level and education. Moreover, the prevalence of MetS was 31% lower in physically active men than in their inactive counterparts, whereas the corresponding percentage among women was 17% [33].

Another study has noted differences between men and women in the association between physical activity or fitness and MetS [34]. Brien et al. [6] reported a significant association between physical activity and MetS in men, but not in women, after adjusting for age, smoking, alcohol consumption and income level. The authors attributed these sex differences to hormonal differences, varying patterns of fat distribution, differences in the definition of MetS, and differences in the types and intensities of physical activity [8,34]. Therefore, the results of the present study and those of other investigations may represent a true difference between sexes in terms of the association between physical activity and MetS.

### Cutoff value for the required amount of physical activity to prevent the onset of MetS

This study also aimed to establish a cutoff value for the required amount of physical activity that would prevent MetS in middle-aged Japanese individuals. Although many studies have included cross-sectional analyses [6,9,13,30,31], our study was the first, to our knowledge, to examine the optimal cutoff value of physical activity required to prevent the onset of MetS. CART analysis revealed that the optimal cutoff value of 26.5 METs h/week represented the level of physical activity required to prevent MetS, and was slightly higher than the value reported by EPAR2006 (23 METs h/week) to prevent lifestyle-related disease [14,15].

Furthermore, applying the EPAR2006 physical activity recommendations resulted in a MetS prevalence of 41.0% (data not shown), which was much higher than that determined in our study (25.4%) when we applied the new cutoff value for physical activity (Figure 1). These results suggest that the EPAR2006 recommendations for physical activity should be increased to reduce the prevalence of MetS among middle-aged Japanese individuals. Our results do not negate the current EPAR2006 physical activity reference value, and the discrepancy between the physical cutoff values can be explained. First, the EPAR2006 physical activity reference was established using a systematic review that primarily summarized the evidence on the effects of physical activity on lifestyle-related disease, rather than focusing on MetS. Second, this systematic review included studies performed in Western countries, and relatively few studies in Japanese individuals were included. Third, the techniques used to measure physical activity may have differed. However, the present study established an association between objectively measured physical activity and MetS in Japanese individuals. For these reasons, the findings of this study might have an important influence on effective promoting physical activity focused on preventing MetS among middle-aged Japanese individuals.

### Strengths and limitations

The primary strength of our study was that we used a triaxial accelerometer to objectively measure physical activity and sedentary times. However, there are several limitations to be discussed. First, it is not possible to infer causality or specify the direction of the observed effects because of the cross-sectional study design. Second, prevalence of MetS/pre-MetS was higher in this study subjects (men: 53.1%, women: 34.2%) compared with general Japanese adults in the National Health and Nutrition Examination Survey in 2008 (men: 45.3%, women: 18.6%). Therefore, the results in this study may not generalize to the Japanese population. Although we controlled for several covariates, there may be other unexplored residual confounding variables such as genetic variation and sociocultural factors that may partly explain our findings. Nevertheless, we considered factors directly related to MetS prevalence, including sedentary time, low intensity physical activity, calorie intake, smoking, menopause status and BMI [35-38], in this study. The study sample size may also be a limitation, but it was sufficiently large to allow us to perform CART analyses to determine the optimal cutoff value for physical activity necessary to prevent MetS. Despite these limitations, our findings help us to understand the association between physical activity and MetS in middle-aged Japanese individuals.

## Conclusions

In conclusion, the results of this study indicate that the EPAR2006 physical activity reference value was associated with MetS/pre-MetS in men. Moreover, we found that moderate physical activity of > 26.5 METs h/week may decrease the risk of MetS in middle-aged Japanese individuals. The validity of the cutoff value for physical activity and the efficacy of physical activity programs that focus on the factors described here to prevent and treat MetS will need to be confirmed in longitudinal and interventional studies.

## Competing interests

The authors declare that they have no competing interests.

## Authors' contributions

JK helped design the study, was responsible for data collection and analysis, and helped to write the manuscript. KT and NY transformed the accelerometer data and provided advice on statistical analyses. HZ helped to measure physical activity. SK was jointly responsible for the concept and design of the study. All authors read and approved the final version of the manuscript for publication.

## Pre-publication history

The pre-publication history for this paper can be accessed here:

http://www.biomedcentral.com/1471-2458/11/624/prepub

## References

[B1] Reaven GM Role of insulin resistance in human disease Diabetes 1998 37 1595 1607 10.2337/diab.37.12.1595 3056758

[B2] Laaksonen DE Lakka HM Niskanen LK Kaplan GA Salonen JT Lakka TA Metabolic syndrome and development of diabetes mellitus: application and validation of recently suggested definitions of the metabolic syndrome in a prospective cohort study Am J Epidemiol 2002 156 1070 1077 10.1093/aje/kwf145 12446265

[B3] Lorenzo C Okoloise M Williams K Stern MP The metabolic syndrome as predictor of type 2 diabetes: the San Antonio heart study Diabetes Care 2003 26 3153 3159 10.2337/diacare.26.11.3153 14578254

[B4] Lakka HM Laaksonen DE Lakka TA Niskanen LK Kumpusalo E Tuomilehto J Salonen JT The metabolic syndrome and total and cardiovascular disease mortality in middle-aged men JAMA 2002 288 2709 2716 10.1001/jama.288.21.2709 12460094

[B5] Hu G Qiao Q Tuomilehto J Balkau B Borch-Johnsen K Pyorala K Prevalence of the metabolic syndrome and its relation to all-cause and cardiovascular mortality in nondiabetic European men and women Arch Intern Med 2004 164 1066 1076 10.1001/archinte.164.10.1066 15159263

[B6] Brien SE Katzmarzyk PT Physical activity and the metabolic syndrome in Canada Appl Physiol Nutr Metab 2006 31 40 47 10.1139/h05-024 16604140

[B7] Lakka TA Laaksonen DE Physical activity in prevention and treatment of the metabolic syndrome Appl Physiol Nutr Metab 2007 32 76 88 10.1139/h06-113 17332786

[B8] Yang X Telama R Hirvensalo M Mattsson N Viikari JS Raitakari OT The longitudinal effects of physical activity history on metabolic syndrome Med Sci Sports Exerc 2008 40 1424 1431 10.1249/MSS.0b013e318172ced4 18614950

[B9] Rennie KL McCarthy N Yazdgerdi S Marmot M Brunner E Association of the metabolic syndrome with both vigorous and moderate physical activity Int J Epidemiol 2003 32 600 606 10.1093/ije/dyg179 12913036

[B10] Franks PW Ekelund U Brage S Wong MY Wareham NJ Does the association of habitual physical activity with the metabolic syndrome differ by level of cardiorespiratory fitness? Diabetes Care 2004 27 1187 1193 10.2337/diacare.27.5.1187 15111543

[B11] Ekelund U Brage S Franks PW Hennings S Emms S Wareham NJ Physical activity energy expenditure predicts progression toward the metabolic syndrome independently of aerobic fitness in middle-aged healthy Caucasians: the Medical Research Council Ely Study Diabetes Care 2005 28 1195 1200 10.2337/diacare.28.5.1195 15855588

[B12] Laaksonen DE Lindström J Lakka TA Eriksson JG Niskanen L Wikström K Aunola S Keinänen-Kiukaanniemi S Laakso M Valle TT Ilanne-Parikka P Louheranta A Hämäläinen H Rastas M Salminen V Cepaitis Z Hakumäki M Kaikkonen H Härkönen P Sundvall J Tuomilehto J Uusitupa M Finnish diabetes prevention study Physical activity in the prevention of type 2 diabetes: the Finnish diabetes prevention study Diabetes 2005 54 158 165 10.2337/diabetes.54.1.158 15616024

[B13] Méndez-Hernández P Flores Y Siani C Lamure M Dosamantes-Carrasco LD Halley-Castillo E Huitrón G Talavera JO Gallegos-Carrillo K Salmerón J Physical activity and risk of metabolic syndrome in an urban Mexican cohort BMC Public Health 2009 9 276 10.1186/1471-2458-9-276 19646257 PMC2734848

[B14] General Affairs Division Health Service Bureau Ministry of Health Labour and Welfare of Japan Exercise and physical activity reference quantity for health promotion 2006 (EPAR2006)-Physical activity, Exercise, and Physical Fitness http://www.nih.go.jp/eiken/programs/pdf/epar2006.pdf

[B15] Ishikawa-Takata K Tabata I Exercise and Physical Activity Reference for Health Promotion 2006 (EPAR2006) J Epidemiol 2007 17 177 17827865 10.2188/jea.17.177 PMC7058479

[B16] Sallis J Saelens B Assessment of physical activity by self-report: status, limitations, and future directions Res Q Exerc Sport 2000 71 1 14 25680007 10.1080/02701367.2000.11082780

[B17] Shephard RJ Limits to the measurement of habitual physical activity by questionnaires Br J Sports Med 2003 37 197 206 10.1136/bjsm.37.3.197 12782543 PMC1724653

[B18] Donahoo W Levine J Melanson E Variability in energy expenditure and its components Curr Opin Clin Nutr 2004 7 599 605 10.1097/00075197-200411000-00003 15534426

[B19] Harris T Owen C Victor C Adams R Ekelund U Cook D A comparison of questionnaire, accelerometer, and pedometer: measures in older people Med Sci Sport Exer 2009 41 1392 1402 10.1249/MSS.0b013e31819b3533 19516162

[B20] Oshima Y Shiga T Namba H Kuno S Estimation of whole-body skeletal muscle mass by bioelectrical impedance analysis in the standing position Obes Res Clin Pract 2010 4 1 10.1016/j.orcp.2009.06.001 24345620

[B21] Oshima Y Kawaguchi K Tanaka S Ohkawara K Hikihara Y Ishikawa-Takata K Tabata I Classifying household and locomotive activities using a triaxial accelerometer Gait Posture 2010 31 370 374 10.1016/j.gaitpost.2010.01.005 20138524

[B22] Trost SG Pate RR Freedson PS Sallis JF Taylor WC Using objective physical activity measures with youth: how many days of monitoring are needed? Med Sci Sports Exerc 2000 32 426 431 10.1097/00005768-200002000-00025 10694127

[B23] The Examination Committee of Criteria for 'Metabolic Syndrome' in Japan Criteria for 'metabolic syndrome' in Japan J Jpn Soc Int Med 2005 94 188 203 in Japanese

[B24] Fujita T The metabolic syndrome in Japan Nat Clin Pract Cardiovasc 2008 5 15 18 10.1038/ncpcardio0808 18580861

[B25] Breiman LFJ Olshen RA Stone CJ Classification and Regression Trees 1984 Belmont, CA: Chapman & Hall/CRC

[B26] Nakata Y Okura T Matsuo T Tanaka K Factors alleviating metabolic syndrome via diet-induced weight loss with or without exercise in overweight Japanese women Prev Med 2009 48 351 356 10.1016/j.ypmed.2009.01.013 19463489

[B27] R Development Core Team R: A Language and Environment for Statistical Computing. R Foundation for Statistical Computing, Vienna, Austria 2009 http://www.R-project.org ISBN 3-900051-07-0

[B28] Cornier MA Dabelea D Hernandez TL Lindstrom RC Steig AJ Stob NR Van Pelt RE Wang H Eckel RH The metabolic syndrome Endocr Rev 2008 29 777 822 10.1210/er.2008-0024 18971485 PMC5393149

[B29] Carroll S Dudfield M What is the relationship between exercise and metabolic abnormalities? A review of the metabolic syndrome Sports Med 2004 34 371 418 10.2165/00007256-200434060-00004 15157122

[B30] Byberg L Zethelius B McKeigue PM Lithell HO Changes in physical activity are associated with changes in metabolic cardiovascular risk factors Diabetologia 2001 44 2134 2139 10.1007/s001250100022 11793014

[B31] Carroll S Cooke CB Butterly RJ Metabolic clustering, physical activity and fitness in nonsmoking, middle-aged men Med Sci Sports Exerc 2000 32 2079 2086 10.1097/00005768-200012000-00018 11128855

[B32] Irwin ML Ainsworth BE Mayer-Davis EJ Addy CL Pate RR Durstine JL Physical activity and the metabolic syndrome in a tri-ethnic sample of women Obes Res 2002 10 1030 1037 10.1038/oby.2002.140 12376584

[B33] Zhu S St-Onge MP Heshka S Heymsfield SB Lifestyle behaviors associated with lower risk of having the metabolic syndrome Metabolism 2004 53 1503 1511 10.1016/j.metabol.2004.04.017 15536610

[B34] Boule NG Bouchard C Tremblay A Physical fitness and the metabolic syndrome in adults from the Quebec Family Study Can J Appl Physiol 2005 30 140 156 10.1139/h05-111 15981784

[B35] Riccardi G Giacco R Rivellese AA Dietary fat, insulin sensitivity and the metabolic syndrome Clin Nutr 2004 23 447 456 10.1016/j.clnu.2004.02.006 15297079

[B36] Ishizaka N Ishizaka Y Toda E Nagai R Yamakado M Association between cigarette smoking, white blood cell count, and metabolic syndrome as defined by the Japanese criteria Intern Med 2007 46 1167 1170 10.2169/internalmedicine.46.0136 17675764

[B37] McNeill AM Rosamond WD Girman CJ Golden SH Schmidt MI East HE Ballantyne CM Heiss G The metabolic syndrome and 11-year risk of incident cardiovascular disease in the atherosclerosis risk in communities study Diabetes Care 2005 28 385 390 10.2337/diacare.28.2.385 15677797

[B38] Eshtiaghi R Esteghamati A Nakhjavani M Menopause is an independent predictor of metabolic syndrome in Iranian women Maturitas 2009 65 262 266 19962253 10.1016/j.maturitas.2009.11.004

